# In vivo Antimalarial and Liver Function Profiles of Methanol Extract of *Salvia officinalis* (Common Sage) Leaf in *Plasmodium berghei*-Infected Mice

**DOI:** 10.4314/ejhs.v34i4.5

**Published:** 2024-07

**Authors:** Azukaego Thomas Hughs Mokogwu, Kingsley Chukwuka Amaihunwa, Collins O Adjekuko, Enekabokom Nwoke Ekene, Edith Omozefe Okoro, Oyebola G Adeosun, Godwin O Avwioro

**Affiliations:** 1 Department of Medical Laboratory Science, Faculty of Science, Delta State University, Abraka, Nigeria; 2 Department of Biological Sciences, University of Delta, Agbor. Nigeria; 3 Department of Pharmacology, Faculty of Basic Clinical Sciences, College of Medical Sciences, Rivers State University, Nkpolu-orowukwo, Port-Harcourt, Nigeria; 4 Department of Medical Laboratory Science, Faculty of Allied Health Sciences, University of Medical Sciences, Ondo City, Ondo State. Nigeria

**Keywords:** Antimalarial models, Salvia officinalis, malaria, liver function profiles, Plasmodium berghe

## Abstract

**Background:**

Salvia officinalis (Common Sage) plant, is used as herbal medicine. The study was aimed at investigating the antimalarial potential and liver function profiles of the Methanol Extract of Salvia officinalis.

**Methods:**

Mice infected with Plasmodium berghei were treated (p.o) with the extract in the curative, suppressive, and prophylactic antimalarial models at doses of 250mg/kg, 500mg/kg, and 1000mg/kg. The positive control drug used was artemether/lumefantrine (7mg/kg A/L) while the negative control was 10mk/kg of Tween 80.

**Results:**

The curative stage showed a significant (p < 0.001) dose-dependent antiplasmodial effect (of the methanol extract of S. officinalis leaf) compared with the negative control (Group 1). At doses of 250, 500, and 1000mg/kg, the Salvia officinalis extract produced parasite suppression of 37.13%, 57.18% and 66.80% respectively. While the positive control group produced parasite percentage suppression of 74.38%. There was a significant chemo-suppressive effect (p < 0.001) at all doses of the methanol extract of Salvia officinalis leaf. The leaf extracts demonstrated a prophylactic significant (p < 0.001) activity. There was no significant effect (p > 0.05) on packed cell volume at doses of 250 and 500mg/kg while 1000mg/kg body weight showed a significant (p < 0.05) effect. There was a reduction in the level of activity of the enzymes and other parameters in the liver function tests with an increase in the dosage of the leaf extract.

**Conclusion:**

The methanol extract of Salvia officinalis possesses in vivo antiplasmodial activities and could be a lead plant in the development of antiplasmodial agents.

## Introduction

Malaria is an infectious disease that results in a high rate of mortality and morbidity in Sub-Saharan Africa (1). The disease is caused and mostly transmitted by female Anopheles *Plasmodium falciparum* mosquitoes (2). It is a major public health burden with an average of 229 million cases and 625,000 deaths annually (3). Nigeria has the highest challenge, with 25% of the global burden and 27% of the global mortality in 2019 (4,5,6). Malarial infestation not only negatively affects the economy but has an unproductive effect on the population (7, 1). A major drawback in the use of the currently available chemotherapeutic drugs is the drug resistance of the malaria parasites, even the artemisinin-based combination therapies (8). Kaushik et al., (9) and Karamanti et al., (10) noted that natural products from medicinal plants have remained a significant source of novel medication, often resulting in fewer side effects, lower cost, improvement in patients' acceptance, and also more consistency with normal physiological function of the human body.

**Figure 1:**
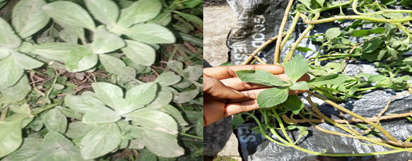
S. officinalis leaves; Source: Photograph of Salvia officinalis leaves taken before drying the leaves

*Salvia officinalis* (Common Sage) plant, a member of the Lamiaceae family is a sumptuous-branched evergreen shrub with a characteristic attractive aroma and a perennial plant used in flavoring species as well as herbal medicine (11,12, 13, 14). The plant is found and cultivated the world over and thrives well in Nigeria, within and around Vom-Jos, Plateau State (Fig. 1) (15). It has been used in the management of cerebral ischemia, memory disorders, depression, lost or declining memory, and Alzheimer's disease (10,16). Common Sage essential oils are indispensable in the management of neurological, heart, metabolic, and endocrine diseases (17, 13,14). The leaf extract of *Salvia officinalis* has been shown to exhibit hepatoprotective, antiplasmin, reducing parasitemia and hepatic oxidative stress in experimental malaria model (18,19). Curative and suppressive tests have been utilized to study the in vivo antimalarial activity of *Penniselum purpureum* leaf (1) while curative, suppressive, and prophylactic tests have been used on the in vivo antiplasmodial activity of methanol leaf extract of *Piliosigma reticulatum* (5). Also, another study employed suppressive, curative, and prophylactic tests to study the in vivo anti-plasmodial activity and toxicological assessment of hydroethanolic crude extract of *Ajuga remote* (20). Some studies (21, 22, 23), have shown that infestation of plasmodium parasites often leads to dysfunction of the liver with resultant elevation of liver enzymes and other biochemical parameters. Therefore, *Salvia officinalis* (Common Sage) plant-wide medicinal application informed the present study titled: In vivo antimalarial and liver function profiles of Methanol Extract of *Salvia officinalis* in *Plasmodium berghei*- Infected Mice utilizing suppressive, curative and prophylactic tests to study its activity.

## Materials and Methods

**Collection, identification and preparation of plant extract**: The *Salvia officinalis* (Sage) plant was collected from Vom-Jos, Plateau State and identified/authenticated by a botanist (Michael, Ozioma Emmanuel) at Delta State University, Abraka (Figure 1). A voucher number (DELSU#134) was assigned to it and stored for future reference. The leaves were cut into small pieces, dried at a temperature of 30 - 40°C for 21 days. The dry leaves were pulverized to obtain a fine powder. 50 g was weighed and added to 400 ml of 95% methanol in a flask. It was shaken at regular intervals for 3 days at room temperature. The mixture was filtered using muslin cloth and concentrated by vacuum evaporator. The extract obtained was kept in an airtight sample bottle, labelled and stored in the refrigerator.

**Animal experiment**: The research was approved by the Research and Ethics Committee, Faculty of Science, Delta State University, Nigeria, and given the reference number REC/DELSU/FOS/2021/02. Adult Swiss mice (male and female) weighing between 20-25g were used in the study. The mice were obtained from the Animal House of the Faculty of Basic Sciences of Delta State University. They were properly housed, and fed with standard growers' marsh and water *ad libitum*. The animal used and handling were done following the International Council for Laboratory Animal Science (ICLAS) and Delta State University Faculty of Science Animal Ethical Committee guidelines. For the extract solution, weighed quantity of *methanol* extract of the *Sage officinalis* leaf (5000 mg/kg) was suspended in distilled water.

**Malaria parasite inoculation**: The blood of the donor Swiss mouse infected with chloroquine-sensitive *Plasmodium berghei* was used for inoculum. *The Plasmodium berghei* (NK-65 strain) was obtained from the Pharmacy Department of Delta State University, Abraka. Each mouse was administered intra-peritoneal (i.p) with 0.2ml of the infected blood containing about 1 x 10^7^ *P. berghei* parasitized red blood cells (standard inoculum). Phosphate-buffered saline (PBS) was used for the dilution of the blood.

**Experimental design**: Twenty-five (25) mice administered (i.p.) with 0.2 ml of standard inoculum were divided randomly into five (5) groups of five (5) mice each (Table 1). Groups 2-4 were treated orally (p.o.) once daily for four days with specific doses of 250, 500 and 1000mg/kg respectively of the methanol extract of *Salvia officinalis* leaf. While group 1(Negative Control) received vehicle only (2% Tween 80 administered at the dose of 10 ml/kg). Group 5 (Positive Control) was treated as standard drug, (7 mg/kg of Artemether/lumefantrine (A/L).

**Table 1:** Experimental design of the work

Group	Number of Mice	Extract / Drug / Vehicle
1	5	10ml/kg of 2% Tween 80
2	5	250mg/kg of Extract
3	5	500mg/kg of Extract
4	5	1000mg/kg of Extract
5	5	7mg/kg Artemether/Lumefartrine (A/L)

**Acute toxicity tests (LD_50_)**: An acute toxicity study (LD_50_) of the methanol extract of the *Salvia officinalis* leaf was conducted in two phases as described by Lorke (24) and reported recently by Evinemi et al.,(1); Ali et al., (25). Nine (9) mice were used in the first phase that comprised of three groups (n = 3). They were given orally, with the methanol extract of the Sage leaf at doses of 10, 100 and 1000mg/kg body weight. Then, the animals were observed for 24 h for signs of toxicity and mortality. The second stage (phase) of three (3) mice (n=1) received specific doses of 1600, 2900 and 5000 mg/kg following the results of the first stage. The lethal dose (LD_50_) was calculated using the formula below:

LD_50_ = √ minimum lethal dose × maximum tolerated dose.

### In vivo Antimalarial tests on the methanol extract of Sage Officinalis

**Grouping and dosing of animals**: *The 4-day suppressive test (Test against early infection)* The Peters (26) method recently adopted by Ali et al., (25) was used. Twenty-five (25) Swiss mice were inoculated (i.p) with 0.2ml of standard inoculums that contain about 1 × 10 *P. berghei*-infected red blood cells. After two (2) hours the animals were grouped into five (5) (n=5) each. The methanol *Sage officinalis* leaf extract was then administered once orally at doses of 250, 500, and 1000 mg/kg to groups 2, 3, and 4 respectively for 4 days. Groups 1 and 5 served as negative and positive controls. Group 1 received 10ml/kg of Tween 80 and Group 5 was given 7mg/kg Artemether/ Lumefartrine (A/L). Four (4) days post-treatment, thin blood films were made on slides from the tail of each mouse. The slides were fixed, stained with Giemsa, and examined for parasite count under the oil immersion objective of the microscope. Three slides were prepared for each mouse and on each slide, three fields were examined to count the red blood cells (RBC). The mean count was taken and the result was used to calculate the percentage (%) parasitemia level. Then, the average parasitemia suppression was calculated using the formula of Tona et al., (27) as shown below:


$$\%\;\text{suppression}=\frac{\%\;\text{parsitaemia}\;\text{in}\;\text{control}-\%\;\text{parsitaemia}\;\text{in}\;\text{treated}\;\text{group}}{\%\;\text{parsitaemia}\;\text{in}\;\text{control}}$$

**Curative antimalarial test (Test against established infection)**: The extract was assessed for its curative ability following the method of Ryler and Peters (28) and used by Ali et al., (25). On day 0, twenty-five (25) mice were given (i.p) with 0.2ml of standard inoculum (containing approximately 1 × 10^7^ *P. berghei* infected erythrocytes). Seventy-two (72) hours postinoculation, the animals were randomly divided grouped into five (5) groups of 5 animals each. Then the animals were treated with the leaf extract at doses of 250, 500, and 1000 mg/kg (orally) in groups 2, 3, and 4 respectively. Groups 1 and 5 which were negative and positive controls received 10 ml/kg of Tween 80 and 7 mg/kg Artemether/Lumefartrine (A/L) respectively. Treatment was done once daily for four (4) days after which thin blood films were made on slides from the tail of each mouse. The slides were fixed with methanol, stained with Giemsa, and examined for parasite count under the oil immersion objective of the microscope. Average parasitemia suppression was calculated using the formula of Tona et al., (27).

**Prophylactic test (Test against residual infection)**: This was done following the residual infection method described by Peters, (26) and recently adapted by Ali et al., (25). Twenty-five (25) Swiss mice were weighted and randomly divided into five groups of five animals each. The Groups 2, 3 and 4 animals were treated with the leaf extract at usual doses of 250, 500, and 1000 mg/kg respectively. Groups 1 and 5 served as negative and positive controls and were treated with 10 ml/kg of Tween 80 and 7 mg/kg Artemether/Lumefartrine (A/L) respectively. The treatment continued daily for four days after which the mice were inoculated with the standard parasite inoculum on the fifth day. Then three days after animals' infestation with the parasite (i.e 72hours post treatment), thin blood films were prepared from each mouse. The slides were fixed with methanol, stained with Giemsa and examined for parasite count under oil immersion objective of the microscope. Average parasitaemia suppression was calculated using the formula of Tona et al., (27).

Before euthanization of the animals, blood was collected directly from tail of each mouse with heparinized capillary tube for packed cell volume measurement and also into heparinized vacutainer for liver function tests.

**Packed cell volume (PCV) measurement**: The PCV was determined by using a micro-hematocrit reader (Hawksley, Finlab) as documented by Bantie et al., (29); Meckonnm (30); Mzena et al., (31) and as reported by Evinemi et al., (1). The heparinized capillary tubes which were ¾^th^ filled with blood, were also sealed at the dry end with sealing clay. The tubes were later placed in a micro-hematocrit centrifuge (Gelma Awhksley, England) with the sealed end outwards and centrifuged at 12,000 rpm for 5 min. The PCV was determined using a standard micro-hematocrit reader.

**Liver function parameters**: Aspartate transaminase (AST) (E.C. 2.6.1.1) and alanine transaminase (ALT) (E.C. 2.6.1.2) by Reitman and Frankel (32), Gamma-glutamyl transferase (GGT) (E.C. 2.3.2.2) by Gjerde and Marland (33), Alkaline phosphatase (ALP) (E.C. 31.3.1) by Kind and King (34), Total proteins (TP) and Albumin (ALB) by Reinhold (35), Total bilirubin (TB) and Conjugated bilirubin (CB) by Malloy and Evelyn (36) methods using Mindray assay kits (Shenzhen China) were measured using an AS-120 Auto-Analyzer (E.LabBST, China: BA-88A).

**Statistical analysis**: Data was analyzed using SPSS, version 21.0. Results obtained were expressed as mean ± standard error of the mean (SEM) and were subjected to ANOVA analysis using Dunnett's post hoc test. A *p*-value of less than 0.05 was considered significant.

## Results

**Acute toxicity test**: The lethal dose (LD_50_) of the methanol leaf extract of *Salvia officinalis* in mice was found to be higher than 5000mg/kg body weight.

**Curative test**: There was a significant (p < 0.001) dose-dependent antiplasmodial effect of the methanol extract of *S. officinalis* leaf compared with the negative control (Group 1). At doses of 250, 500, and 1000mg/kg, the *Salvia officinalis* extract produced parasite suppression of 37.1%, 57.2%, and 66.8% respectively. While the standard drug Artimether/Lumefantrine combination (positive control group) produced parasite percentage suppression of 74.4% (Table 2). The animals in groups 2, 3, and 4 survived longer than the animals in group 1(Negative control). The mice treated with 500 and 1000mg/kg of the leaf extract of *Salvia officinalis* lasted for up to 17 and 21 days respectively compared to group 1 (Negative control) which lasted for only 5 days. Those who were treated with the standard drug (Group 5) lasted for up to 27 days.

**Table 2:** Curative effect of methanol extract of *Salvia officinalis* in *P. berghei*-infected mice

Group	Treatment	% Parasitaemia	Percentage	Mean Survival
			Suppression	Time (Days)
1(Negative Control	10ml/kg (Tween 80)	43.2 ± 0.95	0.00	5.1 ± 1.02
2	250mg/kg Extract	21.8 ± 1.06***	37.1	16.16 ± 1.36
3	500mg/kg Extract	16.0 ± 1.48***	57.2	17.60 ± 0.48
4	1000mg/kg Extract	10.0 ± 0.88***	66.8	19.12 ± 2.32
5(Positive Control)	7mg/kg A/L	06.7 ± 0.72***	74.4	27.14 ± 0.50

Data are presented as mean ± SEM; N = 5: ***P < 0.05 significant

**Suppression test**: The methanol extract of *Salvia officinalis* leaf at doses of 250, 500, and 1000mg/kg body weight showed a significant chemo-suppressive effect (*p* < 0.001) in comparison with group 1 (Negative control). The extract showed a suppressive activity of 24.5%, 56.5%, and 78.4% respectively (Table 3). While the standard drug test showed suppression of 92.0% compared with group 1 (Negative control).

**Table 3:** A 4-day suppressive effect of methanol extract of *Salvia officinalis* in P. berghei-infected mice

Group	Treatment	% Parasitaemia	Percentage
			Suppression (%)
1(Negative Control	10ml/kg (Tween 80)	43.0 ± 1.36	0.0
2	250mg/kg Extract	23.7 ± 1.48***	37.1
3	500mg/kg Extract	16.7 ± 1.86***	57.2
4	1000mg/kg Extract	06.1 ± 0.71***	66.8
5(Positive Control)	7mg/kg A/L	02.3 ± 1.46***	92.0

Data are presented as mean ± SEM; N = 5; ***P < 0.001 significant

**Prophylactic test**: The leaf extract demonstrated significant (*p* < 0.001) activity in comparison with the Tween 80 group (Negative control). Doses at 250, 500, and 1000mg/kg, demonstrated a chemoprophylactic activity of 30.5%, 49.9%, and 79.8% respectively, while the standard drug had a chemoprophylactic activity of 82.7% (Table 4).

**Table 4:** Prophylactic effect of methanol extract of Salvia officinalis in P. berghei-infected mice

Group	Treatment	% Parasitaemia	Percentage
			Suppression (%)
1(Negative Control)	10ml/kg Tween 80	42.6 ± 1.89	0.0
2	250mg/kg Extract	24.8 ± 1.38***	30.5
3	500mg/kg Extract	18.4 ± 2.32***	49.9
4	1000mg/kg Extract	09.9 ± 2.36***	79.8
5(Positive Control)	7mg/kg A/L	06.2 ± 1.24***	82.7

Data are presented as mean ± SEM; N = 5; ***P < 0.001 significant

***Packed cell volume (PCV)****:* The methanol extract of *Salvia officinalis* leaf had no significant effect (*p* > 0.05) on packed cell volume at doses of 250 and 500mg/kg body weight but showed a significant (*p* < 0.05) effect on packed cell volume at dose of 1000mg/kg body weight compared to the Negative control group (Table 5).

**Table 5:** PCV and liver function profiles in the curative stage of methanol extract of *Salvia officinalis* in *P. berghei*-infected mice

Biochemical	10ml/kg T.80	250mg/kg	500mg/kg	1000mg/kg	7mg/kg A/L
parameter	(NC)	Extract	Extract	Extract	(PC)
**PCV (%)**	20.89 ± 1.36	23.14 ± 0.86	26.18 ±1.12	38.31 ± 2.10*	46.19 ± 2.02**
**AST(U/l)**	320.00 ± 2.87	285.10 ± 3.67*	260.72 ± 8.84**	200.34 ± 6.18**	168.72 ± 6.24**
**ALT (U/l)**	147.66 ± 0.43	108.62 ± 1.36*	94.88 ± 2.73*	78.52 ± 2.98**	60.52 ± 2.96**
**GGT(U/l)**	41.16 ± 3.36	36.08 ± 2.18	34.18 ± 3.12*	32.17 ± 2.19**	30.00 ± 3.12**
**ALP(U/l)**	267.36 ± 0.05	185.20 ± 3.72**	171.36 ± 1.21**	158.52 ± 3.72**	146.28 ± 4.16**
**TP(g/l)**	4.33 ± 0.12	4.66 ± 0.30*	4.82 ± 0.18*	4.98 ± 0.40**	5.14 ± 0.12**
**ALB(g/l)**	3.98 ± 0.21	3.74 ± 0.11*	3.70 ± 0.14*	3.62 ± 0.11**	3.60 ± 0.14**
**TB(µmol/l)**	5.44 ± 0.01	5.01 ± 0.01	4.35 ± 0.01*	4.20 ± 0.02*	3.18 ± 0.02*
**CB(µmol/l)**	3.84 ± 0.05	3.58 ± 0.07*	3.20 ± 0.13**	3.05 ± 0.13**	2.58 ± 0.03**

Data are presented as mean ± SEM; N = 5; *P < 0.05, **P < 0.001 significant

PCV = Packed cell volume, AST = Aspartate aminotransferase, ALT = Alanine aminotransferase

GGT = Gamma Glutamyl transferase, ALP = Alkaline phosphatase, TP = Total proteins

ALB = Albumin, TB = Total bilirubin and CB = Conjugated bilirubin

**Liver function parameters**: These tests were done to demonstrate the ability of the leaf-extract to maintain / rebuild the parenchymal activity of the liver cells in parasitaemic disease. There was a dose dependent restoration of the integrity of the parenchymal cells of the liver as demonstrated in the reduction of the level of activity of the enzymes and reduction of the level of other parameters in the liver function tests with increase in the dosage of the leaf extract when compared to the Negative control (Group 1). There was also a corresponding decrease in the level of these liver function parameters on treatment of the mice with the standard drug in comparison with the Negative control group (Table 5).

## Discussions

The acute toxicity tests showed that the methanol extract of *S. officinalis* leaf did not result to any mortality at the employed dose of 5000mg/kg. There was no sign of toxicity; such as skin change, trembling, diarrhea or behavioral changes exhibited by any of the mice. Thus suggesting that the oral LD_50_ of the extract of *S. officinalis* is far greater than the maximum 5000mg/kg body weight, used in the study. Therefore, the extract can be said to be safe in its use for the treatment or management of malarial infection. This finding is in agreement with the work and suggestion of (24, 19, 25) on new approach and safe use of medicinal plants in the management of diseases.

The curative, 4-day suppressive and prophylactic effect of the methanol extract of the Salvia officinalis were utilized in the mice according to the recommendation of (37) and reviewed by Ali et al., (25) who both recommended the in vivo antiplasmodial studies, because such studies consider the possible pro-drug effect and perhaps the involvement of the immune system in the eradication of malarial diseases in humans. Also experimental animals (Mice) and *Plasmodium berghei* were used as suggested by Vinke and Lips (38); Ali et al., (25) based on the parasite sensitivity to chloroquine and antimalarial combination therapy as documented by Builders et al., (39) and currently by Ali et al., (25).

In the curative, 4-day suppressive and prophylactic tests, the methanol extracts of *Salvia officinalis* showed a significant (*p* < 0.001) dose dependent reduction in parasitaemia levels in the study (Tables 2, 3 & 4). This is in agreement with the work of (18, 19) who pointed that Sage is a natural plant that has been used in folk medicine for malarial treatment. Accordingly, they stated that the mechanism of inhibition is probably through formation of a complex between active compounds in leaf of *S. officinalis* and feriheme that prevents the formation of β-hematin. Ali et al., (25) documented that the observed antiplasmodial activity of medicinal plants might be due to the presence of secondary metabolites which could be acting singly or in synergy to exert the observed reduction in parasitemia. However, this is not in doubt as *S. officinalis* is known to be enriched with high concentrations of phytochemicals; tarnins, terpenoids, cardiac glycosides, flavonoids, alkaloids, phenolic, and saponins (15). Equally, the antiplasmodial activity of the extract of *S. officinalis* leaf in this study (Tables 2, 3 & 4) is in tandem with the studies of (40, 41,42) whom all noted that the active phenolic constituents coupled with the aromatic hydroxyl group are responsible for the diverse medicinal characteristics of *S. Officinalis*.

Matthiesen et al., (43) documented that functions of the liver and kidneys are impaired in metabolic disease. In this work, the liver is toxic as shown in the negative control where 10mg/dl of Tween 80 was used for the treatment of *P. berghei-infected* mice (Table 5). The liver toxicity is reflected in the increase in the activity of the aspartate aminotransferase (AST), alanine aminotransferase (ALT), gamma-glutamyltransferase (GGT), alkaline phosphatase (ALP), as well as decrease levels of total proteins (TP), increased levels of albumin (ALB), total bilirubin (TB) and conjugated bilirubin (CB). This finding is in agreement with the works of (21, 22, 23) and the work of (44) who noted that malaria-infected subjects had significantly higher levels of AST, ALT, TB, and CB in their study on Children in Port-Harcourt, Nigeria. Our observation of lower values of albumin (ALB) in the *P. berghei*-infected mice is also similar to their report of lower levels in malarial infected Children in Port-Harcourt. The hyperbilirubinemia could be due to either intracellular hemolysis of the parasitized erythrocytes and micro angiopathic hemolysis often associated with disseminated intravascular coagulation while unconjugated hyperbilirubinemia is a result of massive intravascular hemolysis compared to conjugation which is a hepatocyte dysfunction and hence his association with high activity of the aminotransferases. Also, the observed elevated activity of aminotransferases in our work is in agreement with the work of (21, 22, 23) while our noted increase in ALP is not in agreement with their observed lower ALP. Sumbele et al., (45) and Abdulkadir et al., (5) noted that malarial infection is often associated with lysis of the red blood cells which results in anemia with decreased level of packed cell volume (PCV).

Models such as in vivo tests are often employed in antimalarial studies as they show a possible prodrug effect coupled with improved immunity in the eradication of foreign agents (37). Against early infection in the 4-day suppression study, extract of *S officinalis* most probably reduced the erythrocyte stage development of the *Plasmodium berghei* as parasitemia was suppressed in a dose-dependent approach. This suppressive effect might be due to indirect boasting of the immune system or by the inhibition of the target pathways not known. Also, the induction of parasitemia by the extract of *S. officinalis* might be attributed to the anti-plasmodial activity of specific compounds or a group of compounds (26, 27) such as terpenoids, sterols, flavonoids and saponins as documented by Mokogwu et al., (15). Depiany et al., (48) stated that complicated syndromes of malaria consist of certain inflammatory mediators that may enhance cell to cell interaction and that such cells produce cytokines which produces pyrexia in the host. The antiplasmodial properties of *S. officinalis* extract in both early, established and residual infections may be due to inhibitory effect of cytokines associated compounds as a result of the plant's phytochemical contents. Dysfunction of the liver parenchymal cells that often produce jaundice in a host as a result of infection, involves intravascular hemolysis of erythrocytes coupled with the low immune system as a result of the formation of antigen-antibody complexes to erythrocyte surface. The restoration of this complicated liver dysfunction as shown by the lowering or near normalization of the liver function test parameters perhaps depicts the anti-inflammatory and immunodatory actions of the flavonoids and their associated compounds in the *Salvia officinalis* plant both outside and inside of the host cell (14, 15, 49).

Our finding showed that extracts of *S. officinalis* can prevent hemolysis in malarial-infected mice as depicted by an increase in the various concentrations of the extract and the positive control (7mg/kg A/L) compared to the negative control (10mg/dl Tween 80) infected mice

From our findings, the methanol extract of *Salvia officinalis* showed reasonable antimalarial activities as well as restoration of hepatic injury and could be targeted as a potential lead plant in the development and management of antiplasmodial agents.

## References

[1] Evinemi PA, Enemo K, Onah CM, Uzor PF, Omeje EO (2022). In vivo Antimalarial and GC-MS Studies of Pennisetum purpureum Leaf Extract and Fractions. Trop J Nat Prod Res.

[2] Centers for Disease Control and Prevention (CDC) (2024). United States 2020 Jan. 8.

[3] World Health Organization (2021). WHO World malaria report, 2021.

[4] (2020). WHO Would malaria Report. 20years of global progress and challenges. J. Malar.

[5] Abdulkadir SS, Jatau AI, Abdussalam US, Bichi LA, Abubakar B, Malami S (2022). In vivo antiplasmodial activity of the methanol leaf extract of Piliostigma reticulatum (Dc.) Hochst (Fabaceae). Bulletin of the National Research Centre.

[6] Abdullahi AR, Malami S, Alhassan BL (2021). In vivo antiplasmodial activity of Detarium microcarpum (Fabaceae) stem bark extract. Thrita.

[7] Joy DA, Feng X, Mu J, Furuya T, Chotivanich K, Krettli AU, Ho M, Wang A, White NJ, Suh E, Beerli P, Su XZ (2003). Early origin and recent expansion of Plasmodium falciparum. Sci.

[8] Fairhurst RM, Dondorp AM (2016). Artemisinin-resistant Plasmodium falciparum malaria. Microbiol Spect.

[9] Kaushik NK, Bagavan A, Rahuman AA, Zahir AA, Kamaraj C, Elango G, Jayaseelan C, Kirthi AV, Santhoshkumar T, Marimuthu S, Rajakumar G, Tiwari SK, Sahal D (2011). Evaluation of antiplasmodial activity of medicinal plants from North Indian Buchpora and South Indian Eastern Ghats. Malar J.

[10] Karamati SA, Hassanzadazar H, Bahmani M, Rafieian-Kopaei M (2014). Herbal and chemical drugs effective on malaria. Asian Pac J Trop Dis.

[11] Ayatollahi A, Shojaii A, Kobarfard F, Mohammad zadeh M, Choudhary M (2009). Two flavones from Salvia leriaefolia. Iran J Pharm Res.

[12] Devin SR, Devin KR, Tamizgran M (2021). Sage and treatment of diseases: A mini review. Am J Biomed Sci Res.

[13] Mokogwu ATH, Adjekuko CO, Oshilonyah UH, Ikpefan JO, Eyenubo OB, Avwioro OG (2020b). Hypoglycaemic and Hypolipidemic Effects of Alcoholic Extract of Common Sage (*Salvia Officinalis*) In Streptozotocin-Induced Diabetic Rabbits. Afr J Biomed Res.

[14] Mokogwu ATH, Adjekuko CO, Oshilonyah UH, Ikpefan JO, Avwioro GO (2022a). Antihypertensive and Cardioprotective Effects of *Salvia officinalis* (*Sage*) Leaf in N^G^-Nitro-L-Arginine Methyl Ester (L-NAME) Induced-Hypertensive Wistar Rats. Trop J Nat Prod Res.

[15] Mokogwu ATH, Adjekuko CO, Oshilonyah HU, Avwioro OG (2020). Studies of *Salvia officinalis* leaf extract on some biochemical parameters in rats induced with overdosed-tramadol. Afr J Biotech.

[16] Mohsen AU, Yurttaş L, Özdemir A, Turan-Zitouni G, Kaplancikli ZA (2014). Bazı tetrazol türevlerinin kolinesteraz inhibitörü olarak biyolojik açıdan değerlendirilmesi. Clinical and Experimental Health Sciences.

[17] Khan F, Pallant JF, Amatya B (2011). Outcomes of high- and lowintensity rehabilitation programme for persons in chronic phase after Guillain-Barré syndrome: a randomized controlled trial. J Rehabil Med.

[18] Akkawi M, Sharif AA, Salem K (2012). Wild sage (*Salvia officinalis*) as a potential antimalarial drug. Malar J.

[19] Metwally DM, Alajmi RA, El-Khadragy MF, Al-Quraishy S (2020). Silver Nanoparticles Biosynthesized with Salvia officinalis Leaf Exert Protective Effect on Hepatic Tissue Injury Induced by *Plasmodium chabaudi*. Front. Vet. Sci.

[20] Nardos A, Makonnen E (2017). In vivo antiplasmodial activity and toxicological assessment of hydroethanolic crude extract of Ajuga remota. Malaria Journal.

[21] Ozojiofor UO, Bankole OO, Adedeji IO, Onuh KC (2020). Changes in Liver Function Enzymes in Plasmodium falciparum Infected Malaria Patients in Ajeromi General Hospital, Lagos, Nigeria. Asian Journal of Research in Biochemistry.

[22] Kouam AF, Ngoumé NAN, Fepa AGK, Wainfen Z, Ngounou E, Galani BRT, Nembo NE, Chuisseu PDD, Njayou FN, Moundipa PF (2023). Liver injury in malaria-infected patients in Douala-Cameroon and its association with poor medical practice. Egyptian Liver Journal.

[23] Singh G, Raksha, Urhekar AD, Maheshwari U, Samant P (2018). Role of liver enzymes in patients infected with *Plasmodium vivax* and *Plasmodium falciparium*. Int. J. Adv. Microbiol. Health Res.

[24] Lorke D (1983). A new approach to practical acute toxicity testing. Arch Toxicol.

[25] Ali BH, Mohammed I, Muhammed MA, Mohammed SY, Dauda D, Busola OA, Khadijah II, Manager MM (2022). Evaluation of In vivo Antiplasmodial Activity of the Methanol Root Bark Extract and Fractions of Bombax costatum (Bombacacea) in *Plasmodium berghei*-Infected Mice. Trop. J. Nat. Prod. Res.

[26] Peters W (1965). Drug resistance in *Plasmodium berghei*. Vinka and Lips. Exp Parasitol.

[27] Tona L, Mesia K, Ngimbi NP, Chrimwami B, Okond'Ahoka CK, De Bruyne T, Apers S, Hermans N, Totte J, Pieters L, Vlientick AJ (2001). In-vivo amtimalarial activity of Cassia accidentalis, Morinda morindoides and Phyllanthus niruri. Ann Trop Med Parasitol.

[28] Ryley JF, Peters W (1970). The antimalarial activity of some quinoline esters. Am J Trop Med Parasitol.

[29] Bantie L, Assefa S, Teklehaimanot T, Engidawork E (2014). In vivo antimalarial activity of the crude leaf extract and solvent fractions of Croton macrostachyus Hocsht. (Euphorbiaceae) against *Plasmodium berghei* in mice. BMC Compl Altern Med.

[30] Mekonnen LB (2015). In vivo antimalarial activity of the crude root and fruit extracts of Croton macrostachyus (Euphorbiaceae) against Plasmodium berghei in mice. J Trad Compl Med.

[31] Mzena T, Swai H, Chacha M (2018). Antimalarial activity of Cucumis metuliferus and Lippia kituiensis against *Plasmodium berghei* infection in mice. Res Rep Trop Med.

[32] Reitman S, Frankel S (1957). Colorimetric method for the determination of serum glutamic oxaloacetate and glutamic pyruvate transaminases. Am. J. Clin. Pathol.

[33] Gjerde H, Marland J (1985). Determination of gamma glutamyltransferase in completely haemolysed blood samples. Scandinavian Journal of Clinical and Laboratory Investigation.

[34] Kind PRN, King EJ (1954). Estimation of plasma phosphatase by determination of hydrolysed phenol with 4-amino-antipyrine. Journal of Clinical Pathology.

[35] Reinhold JG, Reiner M (1953). Total proteins, albumin and globulin. Standard Methods of Clinical Chemistry.

[36] Malloy HT, Evelyn KA (1937). The determination of bilirubin with photoelectric photometer. Journal of Biological Chemistry.

[37] Waako PJ, Gumede B, Smith P, Folb PI (2005). The in vitro and in vivo antimalarial activity of Cardiospermum halicacabum L. and Momordica foetida Schumch. Et Thonn. J Ethnopharmacol.

[38] Vinke IH, Lips M (1948). Un nouveau plasmodium d'un rongeursauvage du Congo, *plasmodium berghei* n sp. Ann Soc Belg Med Trop.

[39] Builders MI, Uguru MO, Aguiyi C (2012). Antiplasmodial Potential of the African Mistletoe: Agelanthus dodoneifoliusPolh and Wiens. Indian J Pharm Sci.

[40] Kadhim SU, Mohammed MT, Ahmed OM, Jassim AMN (2016). Study of some *Salvia officinalis* L. (Sage) components and effect of their aqueous extract on antioxidant. International Journal of Chemical Sciences.

[41] El-Feky AM, Aboulthana WM (2016). Phytochemical and Biochemical Studies of Sage (*Salvia officinalis* L.). Pharmaceutical and Biosciences Journal.

[42] Khiya Z, Hayani M, Gamar A, Kharchouf S, Amine S, Berrekhis F, Bouzoubae A, Zair T, Hilali FE (2019). Valorization of the Salvia officinalis L. of the Morocco bioactive extracts: Phytochemistry, antioxidant activity and corrosion inhibition. Journal of King Saud University-Science.

[43] Matthiesen T, Wohrmann T, Coogan TP, Uragg H (1998). The experimental toxicology of tramadol: an overview. Toxicology Letters.

[44] Wokem GN, Nnadi E, Azuonwu O, Okafor A (2018). Effects of Malaria on Selected Liver Function Profiles of Children in Port-Harcourt, Rivers State, Nigeria. International Journal of Tropical Disease & Health.

[45] Sumbele IUN, Kimbi HK, Ndamukong-Nyanga JL, Nweboh M, Anchang-Kimbi JK, Lum E, Nana Y, Ndamukong KKJ, Lehman LG (2015). Malarial anaemia and anaemia severity in apparently healthy primary school children in urban and rural settings in the Mount Cameroon area: cross sectional survey. PLoS ONE.

[46] Muthaura CN, Rukung GM, Chhabra SC, Omar SA, Guantai AN, Gathirwa JW (2007). Antimalarial activity of some plants traditionally used in treatment of malaria in Kwale district of Kenya. J Ethnopharmacol.

[47] Koch A, Tame Z, Pezzato J, Soejarto D (2005). Evaluation of plants used for antimalarial treatment by the Masai people of Kenya. J Ethnopharmacol.

[48] Depinay N, Franetich JF, Grüner AC, Mauduit M, Chavatte JM, Luty AJ (2011). Inhibitory effect of TNF-α on malaria pre-erythrocytic stage development: influence of host hepatocyte/parasite combinations. Plos ONE.

[49] Mali VR, Mohan V, bodhankar SL (2012). Antihypertensive and cardioprotective effects of the Lagenaria siceraria fruit in NG nitro-L-arginine methyl ester (L-NAME) induced hypertensive rats. Pharm Biol.

